# Bee Products: The Challenges in Quality Control

**DOI:** 10.3390/foods12193699

**Published:** 2023-10-09

**Authors:** Qiangqiang Li, Liming Wu

**Affiliations:** State Key Laboratory of Resource Insects, Institute of Apicultural Research, Chinese Academy of Agricultural Sciences (CAAS), Beijing 100093, China; liqiangqiang@caas.cn

## 1. Introduction

In recent years, there has been a significant surge in demand for unprocessed natural foods due to the growing awareness of consumer health. Consequently, both the food and pharmaceutical industries have displayed considerable interest in exploring alternative options to drugs and functional dietary ingredients derived from bee products [1]. Bee products are increasingly acknowledged and embraced by consumers as natural, environmentally friendly products with essential nutritional and therapeutic value. These include honey, bee pollen, propolis, beeswax, royal jelly, bee larvae, queen embryos, etc. The nutritional and bioactive constituents present in these bee products encompass carbohydrates, proteins, peptides, lipids, vitamins, minerals, polyphenols, carotenoids, terpenes, and trace elements. Extensive research has demonstrated that these active functional ingredients confer remarkable antioxidant, antibacterial, anti-inflammatory, immune-regulating, anti-cancer, and tumor-inhibiting properties to bee products. The consumption of these bee products as dietary supplements in various forms, such as tablets, capsules, powders, granules, candy bars, oral liquids, etc., is highly recommended. Furthermore, the vast potential of harnessing these diverse bee products extends across various sectors, including the food, pharmaceutical, and cosmetics industries. However, the utilization of these resources faces multiple obstacles due to challenges such as lack of standardization, limitations in toxicological research, regulations regarding safe consumption dosage, etc.

Furthermore, the remarkable mobility of bees and their direct contact with diverse surfaces facilitate the accumulation of environmental pollutants during foraging through inhalation, ingestion, and adhesion to their body hair. Consequently, these pollutants are subsequently transported to the hive [2]. The presence of potential toxic elements in the environment is often reflected in the composition of bee products such as honey, pollen, and propolis [3]. Bee products are influenced by factors like environmental pollution, residual pesticides, industrial activities, etc., resulting in issues related to residual contamination that pose a threat to consumer health. The investigation into resolutions concerning potential chemical and biological contaminants in bee products, encompassing heavy metals, pesticides, antibiotics, pathogenic microorganisms, etc., can facilitate sustainable development within the apiculture industry while concurrently offering guidance on product safety to ensure consumer health protection.

## 2. Heavy Metal Residues in Bee Products

Due to human activities such as mining, urbanization, agricultural practices, and industrialization, the concentration of chemical pollutants or contaminants in the environment is gradually increasing [4,5]. Bees accumulate heavy metals from the environment through various means such as feeding and body hair. These heavy metals include a range of elements, such as chromium, mercury, manganese, cadmium, lead, arsenic, and silver, among others. Eventually they are transferred to beehives, contaminating bee products like honey, pollen, and propolis [2]. These heavy metals ultimately accumulate or become absorbed into humans and animals, evading complete elimination or degradation, and thereby resulting in adverse health effects [6].

## 3. Residues of Agricultural and Veterinary Drugs

The excessive use and improper application of pesticides and veterinary drugs have led to widespread environmental pollution. These pesticide compounds can also appear in beehives, disrupting bee colony development, and significantly impacting the quality of bee products [7]. Additionally, bees are susceptible to diseases such as American or European foulbrood that can be prevented or controlled by antibiotics. However, the overuse and illegal utilization of these veterinary drugs may result in drug residues being transmitted into bee products, potentially posing health risks to consumers. Long-term ingestion of bee products containing antibiotic residues can lead to antibiotic resistance, mutagenesis, teratogenesis, carcinogenesis, etc. [8].

## 4. Pathogenic Microorganism Contamination

Bee products can be contaminated by various pathogenic microorganisms, directly affecting their safety and quality. Yeasts and a variety of bacteria are present in beehives [9]. Additionally, the intestinal tract of bees contains 70% Gram-negative bacteria (e.g., *Citrobacter*, *Enterobacter*, *Escherichia coli*), 27% Gram-positive bacteria (e.g., *Clostridium* spp., *Streptococcus* spp., *Staphylococcus* spp.), and 1% yeast [10]. The microbial contamination in bee products may originate from beehives, bee intestines, plant flowers, dust, air, etc.; it can also occur during product processing, transportation, and storage processes before being ultimately ingested by consumers [11].

## 5. Potential Allergens in Bee Products

Allergens are also one of the most potential biological food safety risks in bee products. Due to the presence of plant pollen and bee gland proteins in some bee products, allergens may be present, making it easy to cause allergic reactions [12]. The severity of allergic reactions is closely related to the exposure level of allergens and individual physiological conditions, resulting in symptoms that range from mild coughing to anaphylactic shock. The Allergen Database published by the World Health Organization and International Union of Immunological Societies (WHO/IUIS) (http://allergen.org/, accessed on 1 September 2023) has confirmed and registered twelve major allergens from *Apis mellifera*. To improve the safety of consuming bee products, more allergens from bee products like honey, bee pollen, royal jelly, and bee larvae need further exploration.

## 6. Traceability and Authenticity Identification of Bee Products

There are counterfeit products present in the bee product market, which may lack compliant production processes and raise concerns about their quality. Counterfeit products have the potential to mislead consumers and disrupt market order while compromising consumer rights. The quality of bee products is influenced by various factors such as the bee farming environment, floral source quality, processing techniques, etc. It is crucial to establish stringent measures for quality control during collection and production processes in order to enhance product quality management and risk control measures that ensure the authenticity and high standards of bee products. Some bee products might be marketed with specific floral sources or ingredients as selling points without accurate testing or verification procedures being conducted. This discrepancy between product labeling and actual content can lead to confusion among consumers. Therefore, it is imperative to conduct qualitative and quantitative analyses of bee products from both geographical and plant sources perspectives in order to trace their origin. This will involve establishing fingerprint profiles, screening characteristic markers for more precise determination of honey’s authenticity, fostering consumer trust, and providing crucial support for industry development.

This Special Issue aims to publish quality articles on the “Quality Evaluation of Bee Products”. The topics include, but are not limited to, the following:

- Distribution of different nutrients in bee products;
- Residue detection of hazardous substances in bee products;
- Characterization of botanical or geographical markers in bee products;
- Identification of genomic characteristics in bee products;
- Evaluation of biological/functional activities of bee products;
- Application of omic technologies to the composition analysis of bee products.

## References

[1] Fardet A. (2016). Minimally processed foods are more satiating and less hyperglycemic than ultra-processed foods: A preliminary study with 98 ready-to-eat foods. Food Funct..

[2] Finger D., Torres Y.R., Quináia S.P. (2014). Propolis as an indicator of environmental contamination by metals. Bull. Environ. Contam. Toxicol..

[3] Tutun H., Aluç Y., Kahraman H.A., Sevin S., Yipel M., Ekici H. (2022). The content and health risk assessment of selected elements in bee pollen and propolis from Turkey. J. Food Compos. Anal..

[4] Atamaleki A., Sadani M., Raoofi A., Miri A., Bajestani S.G., Fakhri Y., Heidarinejad Z., Khaneghah A.M. (2020). The concentration of potentially toxic elements (PTEs) in eggs: A global systematic review, meta-analysis and probabilistic health risk assessment. Trends Food Sci. Technol..

[5] Ullah R., Jan F.A., Gulab H., Saleem S., Ullah N. (2022). Metals contents in honey, beeswax and bees and human health risk assessment due to consumption of honey: A case study from selected districts in Khyber Pakhtunkhwa, Pakistan. Arch. Environ. Contam. Toxicol..

[6] Solayman M., Islam M.A., Paul S., Ali Y., Khalil M.I., Alam N., Gan S.H. (2016). Physicochemical properties, minerals, trace elements, and heavy metals in honey of different origins: A comprehensive review. Compr. Rev. Food Sci. Food Saf..

[7] O’Neal S.T., Anderson T.D., Wu-Smart J.Y. (2018). Interactions between pesticides and pathogen susceptibility in honey bees. Curr. Opin. Insect Sci..

[8] Okocha R.C., Olatoye I.O., Adedeji O.B. (2018). Food safety impacts of antimicrobial use and their residues in aquaculture. Public Health Rev..

[9] Vázquez-Quiñones C.R., Moreno-Terrazas R., Natividad-Bonifacio I., Quiñones-Ramírez E.I., Vázquez-Salinas C. (2018). Microbiological assessment of honey in Mexico. Rev. Argent. Microbiol..

[10] Al-Waili N., Salom K., Al-Ghamdi A., Ansari M.J. (2012). Antibiotic, pesticide, and microbial contaminants of honey: Human health hazards. Sci. World J..

[11] Orantes-Bermejo F.J., Pajuelo A.G., Megías M.M., Fernández-Píñar C.T. (2010). Pesticide residues in beeswax and beebread samples collected from honey bee colonies (*Apis mellifera* L.) in Spain. Possible implications for bee losses. J. Apic. Res..

[12] Aguiar R., Duarte F.C., Mendes A., Bartolomé B., Barbosa M.P. (2017). Anaphylaxis caused by honey: A case report. Asia Pac. Allergy.

